# Composition and Food Web Structure of Aphid-Parasitoid Populations on Plum Orchards in Chile

**DOI:** 10.3390/insects14030288

**Published:** 2023-03-15

**Authors:** Jeniffer K. Alvarez-Baca, Xiomara Montealegre, Armando Alfaro-Tapia, Francisca Zepeda-Paulo, Joan Van Baaren, Blas Lavandero, Cécile Le Lann

**Affiliations:** 1 Laboratorio de Control Biológico, Instituto de Ciencias Biológicas, Universidad de Talca, Talca 3460000, Chile; 2 ECOBIO (Écosystèmes, Biodiversité, Évolution)-UMR 6553, Université de Rennes 1, CNRS, 6553 Rennes, France; 3 Centro Regional de Investigación e Innovación para la Sostenibilidad de la Agricultura y los Territorios Rurales, Centro Ceres, Pontificia Universidad Católica de Valparaíso, Quillota 2260000, Chile; 4 Instituto Interdisciplinario para la Innovación -I3-, Universidad de Talca, Talca 3460000, Chile

**Keywords:** host-parasitoid interactions, network structure, cover crops, functional ecology, agricultural diversification

## Abstract

**Simple Summary:**

In terrestrial natural ecosystems, more complex and diverse networks of plant–insect primary consumers and their predators are often more productive, stable, and resilient. Plant diversity often positively correlates to the diversity of phytophagous insects and their natural enemies generating multitrophic interactions with changing outcomes (bottom-up effects). The use of cover crops can promote natural enemy populations and their temporal synchronization with a target pest, resulting in greater pest control. Therefore, changes in the habitat conditions can alter food webs. In agroecosystems, characteristics of the food trophic webs, as connectance, measured as the proportion of realized links in the network, could be linked to the efficiency of pest control. In this study, we evaluated how the use of oat cover crops affects composition and structure in the aphid–parasitoid–hyperparasitoid food webs of plum orchards with different habitat management contexts: plums with inter-rows of oats as a cover crop (OCC) and plums with inter-rows with spontaneous vegetation (SV). Quantitative food web metrics differed significantly among treatments showing a higher generality, vulnerability, interaction evenness, and linkage density in SV, while OCC presented a higher degree of specialization.

**Abstract:**

By increasing plant diversity in agroecosystems, it has been proposed that one can enhance and stabilize ecosystem functioning by increasing natural enemies’ diversity. Food web structure determines ecosystem functioning as species at different trophic levels are linked in interacting networks. We compared the food web structure and composition of the aphid– parasitoid and aphid-hyperparasitoid networks in two differentially managed plum orchards: plums with inter-rows of oats as a cover crop (OCC) and plums with inter-rows of spontaneous vegetation (SV). We hypothesized that food web composition and structure vary between OCC and SV, with network specialization being higher in OCC and a more complex food web composition in SV treatment. We found a more complex food web composition with a higher species richness in SV compared to OCC. Quantitative food web metrics differed significantly among treatments showing a higher generality, vulnerability, interaction evenness, and linkage density in SV, while OCC presented a higher degree of specialization. Our results suggest that plant diversification can greatly influence the food web structure and composition, with bottom-up effects induced by plant and aphid hosts that might benefit parasitoids and provide a better understanding of the activity, abundance, and interactions between aphids, parasitoids, and hyperparasitoids in plum orchards.

## 1. Introduction

In terrestrial natural ecosystems, more complex and diverse networks of plant–insect primary consumers and their predators are often more productive, stable, and resilient [1], as the more heterogeneous resources are, the more niches will be available for its exploitation by consumers [2,3]. Therefore, greater heterogeneity and resource quality should increase species coexistence [4]. Plant diversity often positively correlates to the diversity of phytophagous insects and their natural enemies generating multitrophic interactions changing outcomes (bottom-up effects) [5,6,7], by modifying plant-phytophagous-natural enemy networks [8,9,10]. By increasing plant diversity in agroecosystems, it has been proposed that the ecosystem’s functioning can be enhanced and stabilized by increasing natural enemies’ (parasitoids and predators) diversity [8,10,11,12]. Cover crops as well as other habitat management strategies, such as adding flower strips within and around the fields [13,14] or by allowing spontaneous vegetation (SV) growth around the target crop plant [15,16] can be used to benefit natural enemy populations in annual and/or perennial systems [17,18]. Increased plant diversity enhances natural enemy fitness by different mechanisms such as providing shelter from deleterious environmental factors, offering alternative host/prey species, and/or other food resources such as nectar, pollen, and honeydew [19,20,21,22]. As plant resources are generally limited in most agroecosystems, subsequent agricultural diversification can offer more resources that would translate to efficient and numerous natural enemies generating more successful pest control [23,24]. However, increased plant diversity in general does not always favor pest suppression. Indeed, neutral, and even negative, effects of natural enemy diversity on the abundance of pests have been observed [25,26]. Increasing plant diversity may also increase potential negative interactions like intraguild predation or hyperparasitism, resulting in lower pest suppression [25,26,27,28]. It is necessary to better understand the effects of increasing plant diversity on ecosystem functioning by quantifying the functional role of the interactions within insect communities to evaluate the habitat provisioning role in biological control [27,29,30,31].

From an ecological perspective, interacting forces shaping food web structure, such as top-down (predator–prey) and bottom-up (resource–prey–predator) are fundamental to understanding ecosystem functioning [3,32]. In many ecosystems, species are immersed in complex web interactions from antagonistic to mutualistic relationships [5,8,11,13] by interacting directly through competition for resources and indirectly through natural enemies [33]. A way to characterize network interactions among different trophic levels is by using food web metrics which provide an overview of ecosystem structure and functioning [9,34,35]. The most common metrics used for studying herbivore-parasitoid food webs are connectance (which is the proportion of realized links in a food web), linkage density (number of links per species), generality (number of hosts per parasitoids species), vulnerability (number of parasitoids per host species), interaction evenness (the dominance of host-parasitoid interactions within the web) and the specialization metrics at the network level using a web specialization index (H2) as suggested by previous studies (e.g., [36]). In particular, connectance has been used as a good descriptor to show an increase in the food web complexity [37]. When optimized in a rich community, interaction-type diversity can stabilize community dynamics, and diversity of interactions is expected to increase connectance [38]. Biological control should be improved when the connectance between interaction types with reduced herbivore fitness is greater [39]. Therefore, to enhance pest control via natural enemies at the field scale, assessing the composition and structure of insect pest-natural enemy networks is needed for this strategy to be successful [11,40].

Nevertheless, the food web composition of agricultural insect pests and its structure has not often been linked to ecosystem service in the context of plant diversification in agroecosystems. Primary parasitoids and their aphid hosts constitute an interesting system for food web studies due to their close association, as it is possible to determine the host from which an adult parasitoid emerges [41,42], making the measuring of their trophic interactions easier than with other natural enemies [32]. However, hyperparasitoids (i.e., secondary parasitoids of primary parasitoids) should be considered as part of the interacting network as they may disrupt biological control [43,44,45,46]. Studies performed using a food web approach on aphids–parasitoids–hyperparasitoids in cereals have found that food webs were more complex in simple landscapes with lower plant diversity compared to complex and more diverse landscapes [8,43,44]. Likewise, a recent study compared the effect of resource diversification with leguminous intercropping in wheat crops on food web complexity and even when they found no differences in the evaluated metrics (e.g., connectance, generality, etc.), they highlight the importance to consider each specific system including all trophic interacting levels [27]. On the other hand, little is known on orchards as aphids–parasitoids–hyperparasitoids have barely been addressed in a food web context. For instance, the aphid–parasitoid–hyperparasitoid food web composition in citrus has been described where *Aphis spiraecola* (Patch, 1914) (Hemiptera: Aphididae) as the main pest, and *Binodoxys angelicae* (Haliday, 1833) (Hymenoptera: Braconidae) is the dominant primary parasitoid attacked by a complex of hyperparasitoid species, disrupting the biological control [47].

In a previous study, we determined that the provision of an oat cover crop (OCC) within a plum orchard induced an early arrival of natural enemies (parasitoids and coccinellids mainly) by providing alternative aphid hosts. However, we found no evidence of an increased top-down regulation when compared to a treatment where SV was allowed to grow [48]. Nevertheless, we did observe a reduction of the aphid populations on plum trees when the OCC was sown [48]. We also found that oat inter-rows presented less species of aphids, but with a greater aphid abundance (both the densities of oats and aphids were more abundant), whereas the total parasitoid abundance was similar in the inter-rows in both treatments. To better understand the possible mechanisms behind the lack of top-down regulation, in the present study, we compared the composition and structure of the aphid–parasitoid–hyperparasitoid network on plum trees in spring between the two treatments: plum trees with an OCC and plum trees without cover crop but with spontaneous vegetation (SV). As we previously observed more species of aphids in the SV treatment [48], using food web metrics, we hypothesized that (i) the composition of the food web varies between these treatments, with a more diverse assemblage of parasitoids in SV as compared to the OCC treatment and (ii) a higher connectance (possible links for each host-parasitoid pair species) on the SV than on the OCC treatment. Likewise, (iii) a greater mean number of aphids used by each parasitoid species (generality) as well as a greater number of parasitoid species attacking each aphid species (vulnerability) on the SV as compared to the OCC treatment should be observed. In addition, (iv) a higher number of interactions (linkage density) (and a higher network complexity) in the SV treatment than the OCC, which would explain why we previously found no differences in parasitism rates, as higher complexity could favor negative interactions (such as hyperparasitism and competition among species). As we found less aphid species in oat inter-rows but with greater abundances than in SV [48], we also expect that the (v) network specialization (H2) will be higher in the OCC treatment, when compared to the SV treatment, as we expect the dominance of a few parasitoid species and less hyperparasitoids (interaction evenness).

## 2. Materials and Methods

### 2.1. Study Site

This study was located in the central valley of Chile, in Codegua situated in the O’Higgins region (34°08′ S; 70°38′ W). Four farms of organic plums *Prunus domestica* L. (Rosaceae) cv. ‘D’Agen’ with similar management and age were selected. No synthetic fertilizers or insecticides were used. Each farm was at least 10 ha. The central valley of Chile is characterized by a temperate Mediterranean climate, with dry summers and mild, rainy winters [49]. The mean minimum and maximum temperatures vary between 3° to 13 °C from June–August (winter) and between 25° to 35 °C from September–March (spring–summer seasons) [49,50,51].

### 2.2. Experimental Design

Two treatments were established in each of the four farms (N = 4): the OCC treatment, comprising four consecutive inter-rows of oat, *Avena sativa* L. (Poaceae) of at least 100 m long. The inter-rows were sown during the second week of May in autumn, and the treatment without oats, corresponds to four inter-rows with SV consisting of naturally occurring plants cut with a rotary cutter every two weeks. Each replicate consisted of a plot of 1 ha, resulting in a total of eight plots. To avoid interaction between treatments, the treatments at each plum farm were established at least 10 rows away (about 50 m) (for more detailed information see [48]). Prior to the beginning of the experiments, it was determined that all the aphid species attacking oat and other wild gramineous plants were not registered as attacking and damaging the plum tree shoots [52,53].

### 2.3. Insect Sampling

Sampling was carried out over eight sampling dates during 2018 from July to the end of November. Aphids and aphid parasitoids were collected monthly during winter (three sampling dates: 1: 10 July, 2: 8 August, and 3: 9 September) and every two weeks during spring (five sampling dates: 4: 25 September, 5: 9 October and 6: 23 October, 7: 6 November and 8: 20 November) to accurately record the aphid colonization and breakdown that normally occur at this time in plum orchards [54]. All living and parasitized aphids (i.e., mummies) [55] were collected on 20 randomly selected trees, then we chose 20 randomized shoots/tree. Living aphids were taken back to the laboratory, separated, and counted. Collected aphid mummies were individualized in 1.5 mL plastic Eppendorf ^TM^ PCR clean microcentrifuge tubes Thermo Fisher^®^, Waltham, MA, USA (with a small hole in the tip to let air pass) until adult parasitoid emergence in the laboratory. Emergence was checked once daily. Additionally, potentially parasitized but still living aphids were kept on hydrated plum leaves until possible mummy formation. Once formed, mummies were isolated in Eppendorf tubes until emergence. All living samples (mummies and parasitized aphids) were maintained under controlled conditions in climatic chambers (20 ± 1 °C, 65 ± 10% RH, and 16 L: 8 D). Aphids were determined to species level, following taxonomic keys [52,53,56]. After their emergence, parasitoids were identified using taxonomic keys [57,58] and hyperparasitoids were identified to the genus level [59]. Non-emerged individuals were preserved in 95% ethanol for further molecular identification. The species’ richness and diversity of parasitoid species were registered using the Shannon and Simpson indices, as well as the Evenness of Pielou index (see data analysis). They were calculated for each plot (total season) for both treatments.

### 2.4. Non-Emerged-Primary Aphid Parasitoids and Hyperparasitoids Identification

#### 2.4.1. DNA Extraction and Quantification

Non-emerged individuals inside the mummies were dissected to corroborate the presence of a single primary parasitoid or a hyperparasitoid individual. Once dissected, each individual was taken out of the mummy for species identification through DNA extraction. The total DNA of each sample was extracted using a “cells and tissues” DNA isolation kit (Norgen Biotek Corp., Thorold, ON, Canada) following the manufacturer’s instructions with some modifications: The incubation time of the Lysate was 24 h at 37 °C and then 1 h at 56 °C. This was done to obtain the most DNA due to the very small size of the samples. Then, when the clean DNA was obtained, we set up an incubation room temperature time of 5 min to ensure a better elution of the DNA sample. The quantification of the extracted DNA was examined by optical absorbance using an Epoch microplate spectrophotometer (BioTek Instruments, Winooski, VT, USA).

#### 2.4.2. PCR Amplification

A fragment of the COI gene was amplified from the parasitoid DNA using the universal invertebrate primers LCO-1490 and HCO-2198 (Folmer primers) [60]. A PCR amplification was performed with a final volume of 25 µL containing 15 µL of the master mix, which included 0.25 µL Taq DNA polymerase (5 U/µL), 2.5 μL of buffer 1X, 0.2 mM dNTP, 3 mM MgCl_2_, 8.25 µL of ddH_2_O, 10 µL DNA, and 1 µM of each primer plus. The PCR cycling profile was as follows: An initial heating period of 95 °C for 4 min, followed by 36 cycles of 94 °C for 45 s, 50 °C for 45 s, and 72 °C for 1 min; 1 cycle of 72 °C for 5 min; and a final extension step of 72 °C to infinity. PCR products were stored at 4 °C until their visualization in 1.5% agarose gel using 100 V for 60 min.

#### 2.4.3. Sequencing and Editing

All PCR products were sent to Macrogen Inc. (Seoul, Republic of Korea) for purification and bidirectional sequencing to obtain a region of 600 bp approximately, of the barcoding region of the COI gene. Once obtained, the DNA sequences (forward and reverse) were edited using BioEdit sequence alignment editor v7.2.5 [61] to generate a consensus sequence for each sample. Finally, the sequences were compared and analyzed by BLASTn on NCBI (https://www.ncbi.nlm.nih.gov/, accessed on 2 June 2021) and the Barcode of Life Data System v4 (https://www.boldsystems.org/, accessed on 5 June 2021). The species were determined by the similarity of the alignment ≥98% of the sequences of the individuals in both libraries. The species identification was confirmed with DNA sequences of adult parasitoids obtained from the same sampling field (Lavandero et al., unpublished data). Due to problems with the sequencing, five individuals could not be identified to the species level and were thus discarded from the analysis.

### 2.5. Quantitative Food Web Construction

Two steps were considered to construct the food webs: First, two trophic levels considering the aphid species–primary parasitoids interactions and, second, two trophic levels considering the interactions among aphid species–hyperparasitoids. But aphid–parasitoid–hyperparasitoid food webs in this study were not considered, as the identity of the primary parasitoids was unknown when hyperparasitoids were the emerging adult species. Food webs were constructed for each treatment considering all interactions over spring (all dates pooled) based on the relative abundance of aphids with known links to a parasitoid species, and their emerged parasitoids and hyperparasitoids species including specimens without adult emergence from mummies, but that were identified to the species level with molecular markers (see above) collected to show the degree of links between them. The trophic interaction networks were plotted using the Food Web Designer v3.0 software [62].

### 2.6. Food Web Metrics

Quantitative network metrics: Generality, the weighted mean number of hosts per parasitoids species; Vulnerability, the weighted mean number of parasitoids per host species; Connectance, the realized proportion of possible links observed in a food web; H2, the level of specialization within a network (0 = no specialization to 1 = perfect specialization). Weighted linkage density, the mean number of links per species weighted by the number of interactions and Interaction evenness, the dominance of specific host-parasitoid interactions within the web (1 = interactions equally represented, <1 = some interactions more dominant than others) were calculated per treatment for all aphid specimens with parasitoid or hyperparasitoid emergence (via taxonomical or molecular identification) [32,63,64].

### 2.7. Statistical Analysis

Quantitative food web metrics were built using the network level function from Bipartite R package [65] for each treatment during all five sampling dates to show the composition of the aphid–primary parasitoids as well aphid–hyperparasitoids assemblages. The richness, Shannon–Wiener and Simpson diversity indices, and the Evenness of Pielou index of parasitoid species were calculated for each plot on both treatments using the vegan package in R package [66]. The effect of the treatments (SV and OCC) on the food web metrics (generality, vulnerability, connectance, H2, weighted linkage density and interaction evenness) was evaluated using GLMs assuming a Gaussian distribution and the ‘identity’ link function. The log of the total abundance of the insect individuals was included as a covariate [64] since the quantitative food web metrics are strongly affected by network sizes [41]. Additionally, to evaluate the effect of the treatment on the diversity indices, we performed GLMs assuming a Gaussian distribution and the ‘identity’ link function (Shannon–Wiener, Simpson, and Evenness of Pielou index) and the species richness assuming a Poisson distribution and the “log link” function for counting data. All the analyses were performed using the R package 3.6.5 [67]. Generalized linear models (GLMs) were conducted using the lme4 package [68]. The best model was chosen using the Akaike information criteria (AIC) after performing an ANOVA type II in the car package following a stepwise regression method [69]. Post hoc pairwise comparisons were carried out using Tukey tests, correcting for multiple comparisons with the single-step method using the Multcomp package [70].

## 3. Results

During the winter, we found neither aphid eggs or nymphs or adults, nor parasitoids or hyperparasitoids on the plum trees. During the spring, a similar composition of aphid species was observed in both treatments. A total of 4865 aphids were recorded in the SV treatment, including living aphids and mummies, from which 76.81% corresponded to *Brachycaudus helichrysi* (Kaltenbach, 1843), 19.67% to *A. spiraecola* and 3.51% to *Myzus persicae* (Sulzer, 1776) (Hemiptera, Aphididae). On the other hand, 2752 aphids were recorded in the OCC treatment; out of these, 66.13% corresponded to *B. helichrysi,* 27.94% to *A. spiraecola* and 5.92% to *M. persicae*. From the total number of aphids collected, 215 mummies were recorded in the SV treatment with an emergence rate of 68.84%. From these mummies, 158 corresponded to primary parasitoids and 57 to hyperparasitoids. In the case of the OCC treatment, there were 29 mummies with an emergence rate of 75.86%. From these, 23 individuals corresponded to primary parasitoids and 6 to hyperparasitoids.

**Figure 1:**
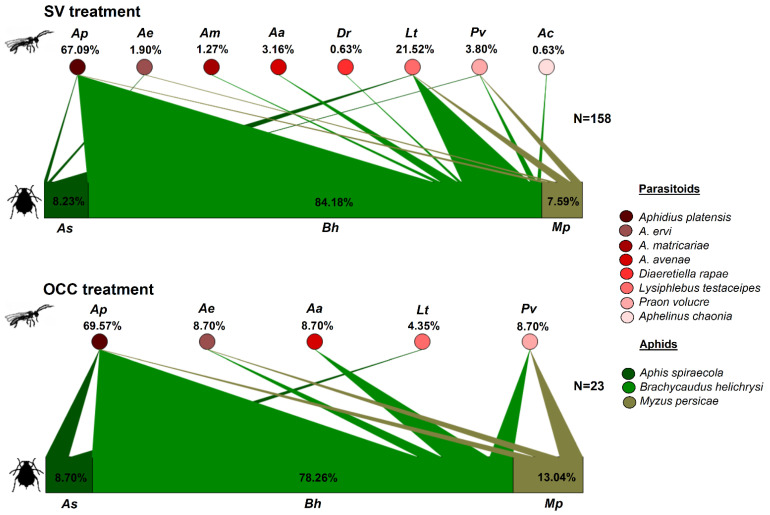
Quantitative food webs of aphids–primary parasitoids during spring in plum orchards in Chile comparing two treatments: Spontaneous vegetation (SV) and oat cover crop (OCC). The horizontal bars represent the relative abundances of each aphid species parasitized (**lower** bars) and their primary parasitoids (**upper** circles). The arrows represent the strength of interaction (% relative abundances) between each aphid host–parasitoid combination. N = corresponds to the number of adult parasitoids emerged in all samples (unparasitized aphids were not considered).

Primary parasitoids composition was similar in both treatments, but with a significantly higher richness of parasitoid species in the SV treatment (mean richness was 6.6 species) in comparison to OCC treatment (mean richness was 2.5 species) (X^2^ = 6.89, *p* = 0.01, Table 1). Although no significant differences were found in the Shannon and Simpson indexes between treatments, OCC presented more dominance than SV (Evenness 0.62 versus 0.42, *p* = 0.05, Table 1). From the total number of mummies recorded in the SV treatment, *Aphidius platensis* (Brethes, 1913) represented 67.09%, followed by *Lysiphlebus testaceipes* (Cresson, 1880) (21.52%), while *Praon volucre* (Haliday, 1833), *Aphidius avenae* (Haliday, 1834), *Aphidius ervi* (Haliday, 1834), *Aphidius matricariae* (Haliday, 1834), *Diaeretiella rapae* (M’Intosh, 1855) (Hymenoptera, Braconidae) and *Aphelinus chaonia* (Walker, 1839) (Hymenoptera, Aphelinidae) accounted for 11.39% (Figure 1). In the OCC treatment, from the total number of mummies, 69.57% corresponded to *A. platensis*, 8.7% each to *P. volucre*, *A. avenae*, *A. ervi* and 4.35% to *L. testaceipes* (Figure 1). It was also observed that *A. platensis* was mainly associated with the common host *B. helichrysi* in both treatments (Figure 1).

**Figure 2:**
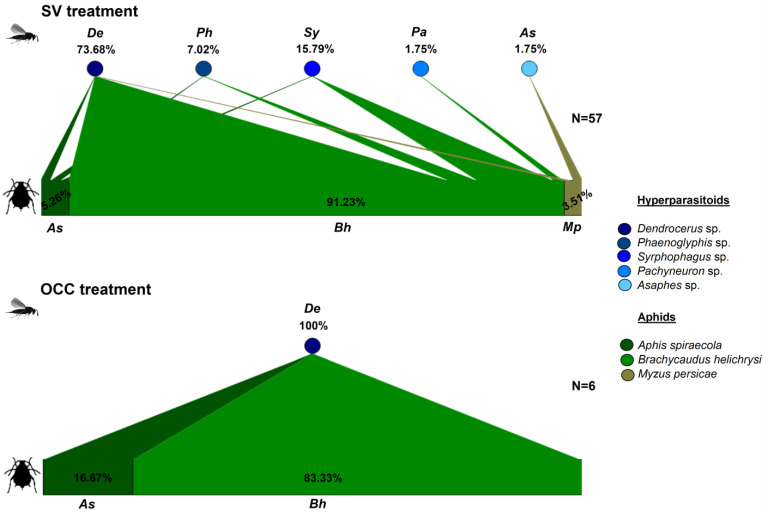
Quantitative food webs of aphids–hyperparasitoids (secondary parasitoids) during spring in plum orchards in Chile comparing two treatments: Spontaneous vegetation (SV) and oat cover crop (OCC). The horizontal bars represent the relative abundances of each aphid species parasitized with hyperparasitoid emergence (**lower** bars) and the hyperparasitoids (**upper** circles). The arrows represent the strength of interaction (% relative abundances) between each aphid host–hyperparasitoid combination. N = corresponds to the number of adult parasitoids emerged in all samples (unparasitized aphids were not considered).

Similarly, hyperparasitoid assemblages differed among treatments, with a higher number of genera in the SV than in the OCC treatment. In the SV treatment, from the total number of mummies collected, 73.68% corresponded to *Dendrocerus* sp. (Ratzeburg, 1852) (Hymenoptera: Megaspilidae), 15.79% to *Syrphophagus* sp. (Ashmead, 1900) (Hymenoptera. Encyrtidae), with the remaining 10.53% corresponding to *Phaenoglyphis* sp. (Förster, 1869) (Hymenoptera: Figitidae), *Pachyneuron* sp. (Walker, 1833), and *Asaphes* sp. (Walker 1834) (Hymenoptera: Pteromalidae) together (Figure 2). While in the OCC treatment, all recorded mummies corresponded only to *Dendrocerus* sp, being highly associated with *B. helichrysi* in both treatments (Figure 2).

**Figure 3:**
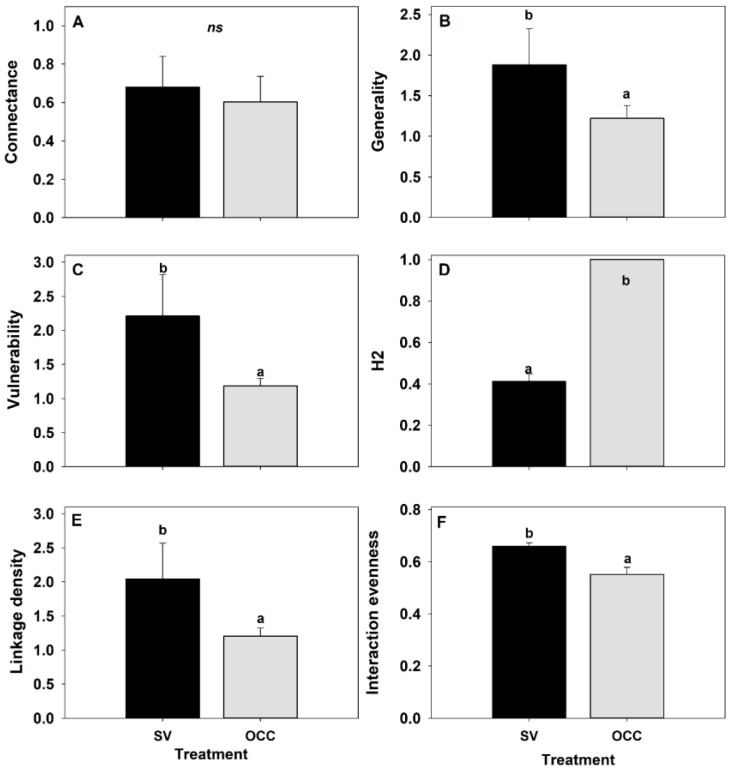
Food web metrics of the plum orchards in Chile with spontaneous vegetation (SV) and with oat cover crop (OCC). (**A**) Connectance, (**B**) Generality, (**C**) Vulnerability, (**D**) H2, (**E**) Linkage density, and (**F**) Interaction evenness. Different letters indicate significant differences between treatments and ‘ns’ indicates non-significant differences.

Finally, for the food web metrics, there were not significant differences for connectance (GLM: ꭓ^2^ = 0.73; df = 1, *p* = 0.39) (Figure 3A) among treatments. By contrast, there were differences among treatments for the remaining metrics: Generality (GLM: ꭓ^2^ = 6.86; df = 1, *p* = 0.009) being higher on the SV compared to the OCC treatment (Figure 3B); a higher vulnerability on the SV than on the OCC treatment (GLM: ꭓ^2^ = 8.56; df = 1, *p* = 0.003) (Figure 3C); a higher specialization (H2) on the OCC than on the SV treatment (GLM: ꭓ^2^ = 216.27; df = 1, *p* < 0.001) (Figure 3D); a higher number of links per species (linkage density) on the SV compared to the OCC treatment (GLM: ꭓ^2^ = 9.27; df = 1, *p* = 0.002) (Figure 3E), and a higher interaction evenness on the SV than on the OCC treatment (GLM: ꭓ^2^ = 9.84; df = 1, *p* = 0.002) (Figure 3E).

## 4. Discussion

In this study, we evaluated how the use of oat cover crops affects the composition and structure of the aphid–parasitoid–hyperparasitoid food webs of plum orchards. Although we found a similar aphid composition between treatments in the plum trees, with similar aphid relative abundances and the specialist aphid *B. helichrysi* as the most abundant aphid species, we found changes in the interactions between aphids and their primary and secondary parasitoids. These observations are in agreement with our first hypothesis, which established that the composition of the food web varies between treatments, with a more diverse assemblage of parasitoids in SV compared to the OCC treatment, showing that the species richness of primary and secondary parasitoids was greater in the SV treatment compared to the OCC. However, the Shannon and Simpson indexes were not significantly different, there was a trend in evenness (Pielou index), with more dominance in the OCC due to the primary parasitoid *A. platensis*. For the hyperparasitoids, the only secondary parasitoid species found was the hyperparasitoid *Dendrocerus* sp. A similar observation has previously been made in our country for wheat parasitoid–hyperparasitoid assemblages [71]. In the SV treatment, a more complex food web composition was found, with the primary parasitoid guild composed of eight parasitoid species, where *A. platensis*, *A. ervi*, *L. testaceipes* and *P. volucre* showed a plastic host-use, as evidenced in the food webs, as we see them attacking several species of aphids. However, the other species recorded were only parasitizing one aphid species. On the other hand, for the parasitoid assemblage found on the OCC treatment, five parasitoid species were found, and as with the SV treatment, *A. platensis* was the most abundant parasitoid and mainly associated with the most common aphid species, *B. helichrysi* (Figure 1). Hyperparasitoids, which represent the fourth trophic level, may have effects on the aphid population by disrupting biological control of the primary parasitoids [45,72,73]. According to the food web composition analysis, a higher resource diversity (primary parasitoid species) was provided by the SV treatment compared to the OCC treatment. This seems to have been beneficial for the hyperparasitoids in the SV as we found a more complex hyperparasitoid guild with species with a high host use, such as *Dendrocerus* sp., *Asaphes* sp., and *Syrphophagus* sp. [74,75], and species with a restricted host use, such as *Phaenoglyphis* sp. and *Pachyneuron* sp. [76] Similar to our results, a recent study found a different hyperparasitoid species composition when more plant resources were added (leguminous plants) intercropped with cereals [27]. This change in parasitoid composition could be explained although the SV treatment had a patchily distributed, non-persistent and vegetational cover (less plant density), but with a higher species richness, being composed mostly of wild gramineous plants and weeds introduced from other plant families, such as Malvaceae and Asteraceae. Therefore, it offers a more complex habitat in terms of resource availability and spatial heterogeneity for aphids [77] and parasitoids could therefore find a greater diversity of hosts and/or host abundance in a more diverse environment, as observed here for the SV treatment with more abundant hosts compared to the OCC treatment. Although the OCC treatment was characterized by only one plant species (oat) with a high and homogeneous coverage it seems to provide a good source of resources (aphids). Moreover, at least seven parasitoid species found on plums in the present study, have also been reported attacking aphid species on the cereal crops, including *A. platensis* (previously reported in Chile as *Aphidius colemani*, [58]), *L. testaceipes*, *P. volucre*, *A. avenae*, *A. ervi*, *A. matricariae* and *D. rapae* [71,78]. Similarly, a previous study carried on plum orchards found that the main parasitoid species attacking cereal aphids was *A. platensis* [79]. However, in relation to the second hypothesis, we did not observe a higher connectance (possible links for each host–parasitoid pair species) on the SV than on the OCC treatment, despite the increased species richness of parasitoids in the food web for the SV treatment. On the other hand, according to our third and fourth hypotheses that posed a greater generality and vulnerability on the SV compared to the OCC treatment and a higher number of interactions (linkage density) and a higher network complexity in the SV treatment than in the OCC, respectively; the quantitative food web analysis showed a greater mean number of aphid species used by parasitoids (generality) and a greater number of parasitoid species attacking aphids (vulnerability) on the SV treatment in comparison to the OCC treatment. Likewise, a higher network complexity and a higher number of interactions (linkage density) were observed on the SV compared to the OCC treatment. Finally, as expected from our fifth hypothesis, network specialization (H2) was higher in the OCC treatment, when compared to the SV treatment. As we expected, the dominance of a few parasitoid species (specifically one parasitoid species *A. platensis* and one hyperparasitoid species *Dendrocerus* sp.), would increase network specialization. These findings suggest that the connectance, measured as the realized proportion of potential link density, was more related to the dominance of *A. platensis* in both treatments across the study. This could be explained since this species of parasitoid might benefit from the availability of the common and prevalent host resources from both treatments [80,81], where most parasitoids were connected in a single large compartment [82]. Likewise, the higher generality in the SV treatment may be the result of a bottom-up effect of the more diverse spontaneous plant–aphid resources on the aphid–parasitoid interactions. Within our study, the food web composition, structure, and density of inter-rows vegetation changed between treatments: more diverse in the SV treatment and more homogeneous in the OCC treatment, allowing primary parasitoids to exploit more aphid hosts in the SV compared with the OCC treatment. This pattern of a trophic cascade (plant–aphid host–parasitoid) is very common in aphid–parasitoid systems [81]. Therefore, the SV exerted strong effects on parasitoid diversity and food web generality. Similarly, the difference in habitat complexity between treatments influenced the mean number of parasitoids per aphid species (vulnerability), which could be due to the higher parasitoid richness and their higher relative abundance on the SV treatment compared to the OCC treatment. However, in a previous study we did not find differences in the total parasitism among treatments [48]. Therefore, the higher parasitoid richness on the SV treatment did not translate into a greater aphid control. On the contrary, we found more aphid abundance in the SV treatment compared to the OCC [48].

On the other hand, the degree of specialization tends to increase in the OCC in relation to SV treatment. This could suggest that increased competition among parasitoids may be happening due to the higher species richness of parasitoid species in the SV treatment. In our study, the most abundant parasitoid species (*A. platensis*) was more specialized on the most common aphid host (*B. helichrysi*) in both habitats showing reduced attack rates on other hosts, suggesting that habitat modification can alter the structure of an aphid–parasitoid food web [32]. Host specialization in aphid parasitoids has been shown to affect the biological control they provide, due to a higher capacity of parasitoids to change between different hosts allowing them to persist in the absence of their main host (normally the main pest), and to regulate pest outbreaks in a rapidly changing environment [83]. However, the assumptions we make in the study should be taken with caution due to the low number of parasitoids and hyperparasitoids recorded in the OCC treatment compared to SV. The increase in interaction evenness for the SV treatment suggests that most of the interactions occur between few species. Our results revealed that SV indeed promoted parasitoid evenness but instead of limiting aphids in plum orchards, parasitoids seemed to attack only aphids on the spontaneous weeds. However, it is still unclear how these network changes in a habitat management context impact ecosystem functioning and ecosystem services [84]. Our results differ from a previous study where interaction metrics were significantly higher when fields presented less plant diversity compared to fields with higher plant diversity [44]. However, the number of plant species involved in our study is already too low to draw any significant comparisons.

## 5. Conclusions

To enhance the effects of parasitoids, habitat management programs should include the functioning of host–parasitoid systems as well as parasitoid–hyperparasitoid interactions. Plant diversification affects species interactions and can have notable effects on food web structure, with bottom-up effects induced by aphids that might benefit parasitoids, thus exerting a great influence of primary parasitoids on pest control. Our results suggest that plant diversification can greatly influence the food web structure and composition, with bottom-up effects induced by plant and aphid hosts that might benefit parasitoids and provide a better understanding of the activity, abundance, and interactions between aphids–parasitoids–hyperparasitoids in plum orchards. Bottom-up effects will induce food web changes that propagate to the next trophic level, with higher values of quantitative link density, suggesting that linkage density and interaction diversity were positively influenced by the aphid host. However, in our study, we found no variation in primary parasitism, although less aphid infestation was associated with the use of oats as an intercrop [48]. Other bottom-up mediated effects should also be considered in future studies, such as the release of semiochemicals from cover crops or plant-insect semiochemicals such as B-farnesene, which could also interrupt aphid colonization and/or promote dispersal away from associated crops (such as the plum trees in this system) that could affect aphid–parasitoid interactions and biological control of the main crop.

## Figures and Tables

**Table 1:** Summary of diversity indices between the spontaneous vegetation (SV) and the oat cover crop (OCC) treatments for all parasitoids present. In addition, generalized linear models (GLMs) show statistical differences among treatments for each of the indices evaluated.

	Treatment						
	SV		OCC		Model Values		
Index	Mean	SE	Mean	SE	d.f.	*X* ^2^	*p*-Value
Species richness (S)	6.67	2.96	2.50	0.65	1	6.89	***0.01***
Shannon–Weiner diversity index (H′)	0.91	0.51	0.69	0.24	1	0.19	0.66
Simpson (1-D) index	0.42	0.22	0.41	0.14	1	0.00	0.99
Evenness of Pielou index	0.42	0.09	0.62	0.06	1	3.77	0.05

## Data Availability

The data used and/or analyzed during the current study are available from the corresponding author on reasonable request.

## References

[1] May R.M. (1972). Will a large complex system be stable?. Nature.

[2] Ricklefs R. (1976). Environmental heterogeneity and plant species diversity: A hypothesis. Am. Nat..

[3] Hunter M.D., Price P.W. (1992). Playing Chutes and Ladders: Heterogeneity and the Relative Roles of Bottom-Up and Top-Down Forces in Natural Communities. Ecology.

[4] Wright D.H. (1983). Species-Energy Theory: An Extension of Species-Area Theory. Oikos.

[5] Tylianakis J.M., Laliberté E., Nielsen A., Bascompte J. (2010). Conservation of species interaction networks. Biol. Conserv..

[6] Haddad N.M., Crutsinger G.M., Gross K., Haarstad J., Tilman D. (2011). Plant diversity and the stability of foodwebs. Ecol. Lett..

[7] Moreira X., Mooney K.A. (2013). Influence of plant genetic diversity on interactions between higher trophic levels. Biol. Lett..

[8] Gagic V., Hänke S., Thies C., Scherber C., Tomanović Ž., Tscharntke T. (2012). Agricultural intensification and cereal aphid-parasitoid-hyperparasitoid food webs: Network complexity, temporal variability and parasitism rates. Oecologia.

[9] Thompson R.M., Brose U., Dunne J.A., Hall R.O., Hladyz S., Kitching R.L., Martinez N.D., Rantala H., Romanuk T.N., Stouffer D.B. (2012). Food webs: Reconciling the structure and function of biodiversity. Trends Ecol. Evol..

[10] Roubinet E., Jonsson T., Malsher G., Staudacher K., Traugott M., Ekbom B., Jonsson M. (2018). High redundancy as well as complementary prey choice characterize generalist predator food webs in agroecosystems. Sci. Rep..

[11] Tylianakis J.M., Binzer A. (2014). Effects of global environmental changes on parasitoid-host food webs and biological control. Biol. Control.

[12] Bartomeus I., Gravel D., Tylianakis J.M., Aizen M.A., Dickie I.A., Bernard-Verdier M. (2016). A common framework for identifying linkage rules across different types of interactions. Funct. Ecol..

[13] Balzan M.V., Bocci G., Moonen A.C. (2016). Utilisation of plant functional diversity in wildflower strips for the delivery of multiple agroecosystem services. Entomol. Exp. Appl..

[14] Hatt S., Mouchon P., Lopes T., Francis F. (2017). Effects of wildflower strips and an adjacent forest on aphids and their natural enemies in a pea field. Insects.

[15] Bugg R.L., Waddington C. (1994). Using cover crops to manage arthropod pests of orchards: A review. Agric. Ecosyst. Environ..

[16] Denys C., Tscharntke T. (2002). Plant-insect communities and predator-prey ratios in field margin strips, adjacent crop fields, and fallows. Oecologia.

[17] Karp D.S., Chaplin-Kramer R., Meehan T.D., Martin E.A., De Clerck F., Grab H., Gratton C., Hunt L., Larsen A.E., Martínez-Salinas A. (2018). Crop pests and predators exhibit inconsistent responses to surrounding landscape composition. Proc. Natl. Acad. Sci. USA.

[18] Alarcón-Segura V., Grass I., Breustedt G., Rohlfs M., Tscharntke T. (2022). Strip intercropping of wheat and oilseed rape enhances biodiversity and biological pest control in a conventionally managed farm scenario. J. Appl. Ecol..

[19] Landis D.A., Wratten S.D., Gurr G.M. (2000). Habitat management to conserve natural enemies of arthropod pests in agriculture. Annu. Rev. Entomol..

[20] Pickett C.H., Roltsch W., Corbett A. (2004). The role of rubidium marked natural enemy refuge in the establishment and movement of *Bemisia* parasitoids. Int. J. Pest Manag..

[21] Quijas S., Schmid B., Balvanera P. (2010). Plant diversity enhances provision of ecosystem services: A new synthesis. Basic Appl. Ecol..

[22] Cardinale B.J., Srivastava D.S., Duffy J.E., Wright J.P., Downing A.L., Sankaran M., Jouseau C., Cadotte M.W., Carroll I.T., Weis J.J. (2009). Effects of biodiversity on the functioning of ecosystems: A summary of 164 experimental manipulations of species richness. Ecology.

[23] Gurr G.M., Wratten S.D., Landis D.A., You M. (2017). Habitat management to suppress pest populations: Progress and prospects. Annu. Rev. Entomol..

[24] Hassan K., Pervin M., Mondal F., Mala M. (2016). Habitat Management: A key option to enhance natural enemies of crop pest. Univers. J. Plant Sci..

[25] Letourneau D.K., Jedlicka J.A., Bothwell S.G., Moreno C.R. (2009). Effects of natural enemy biodiversity on the suppression of arthropod herbivores in terrestrial ecosystems. Annu. Rev. Ecol. Evol. Syst..

[26] Poveda K., Gómez M.I., Martínez E. (2008). Diversification practices: Their effect on pest regulation and production. Rev. Colomb. Entomol..

[27] Jeavons E., van Baaren J., Le Ralec A., Buchard C., Duval F., Llopis S., Postic E., Le Lann C. (2021). Third and fourth trophic level composition shift in an aphid–parasitoid–hyperparasitoid food web limits aphid control in an intercropping system. J. Appl. Ecol..

[28] Chaplin-Kramer R., O’Rourke M., Blitzer E., Kremen C. (2011). A meta-analysis of crop pest and natural enemy response to landscape complexity. Ecol. Lett..

[29] Gagic V., Bartomeus I., Jonsson T., Taylor A., Winqvist C., Fischer C., Slade E.M., Steffan-Dewenter I., Emmerson M., Potts S.G. (2015). Functional identity and diversity of animals predict ecosystem functioning better than species-based indices. Proc. R. Soc. B Biol. Sci..

[30] Maisonhaute J.É., Labrie G., Lucas E. (2017). Direct and indirect effects of the spatial context on the natural biocontrol of an invasive crop pest. Biol. Control.

[31] Tylianakis J.M., Morris R.J. (2017). Ecological Networks Across Environmental Gradients. Annu. Rev. Ecol. Evol. Syst..

[32] Tylianakis J.M., Tscharntke T., Lewis O.T. (2007). Habitat modification alters the structure of tropical host-parasitoid food webs. Nature.

[33] Holt D. (1977). Predation, apparent competition, and the structure of prey communities. Theor. Popul. Biol..

[34] Bukovinszky T., Van Veen F.J.F., Jongema Y., Dicke M. (2008). Direct and indirect effects of resource quality on food web structure. Science.

[35] Montoya J.M., Rodríguez M.A., Hawkins B.A. (2003). Food web complexity and higher-level ecosystem services. Ecol. Lett..

[36] Derocles S.A.P., Le Ralec A., Besson M.M., Maret M., Walton A., Evans D.M., Plantegenest M. (2014). Molecular analysis reveals high compartmentalization in aphid-primary parasitoid networks and low parasitoid sharing between crop and noncrop habitats. Mol. Ecol..

[37] van Altena C., Hemerik L., de Ruiter P.C. (2016). Food web stability and weighted connectance: The complexity-stability debate revisited. Theor. Ecol..

[38] Mougi A., Kondoh M. (2012). Diversity of interaction types and ecological community stability. Science.

[39] Kondoh M., Mougi A., Moore J., De Ruiter P., McCann K., Woltors V. (2017). What kind of interaction-type diversity matters for community stability?. Adaptive Food Webs: Stability and Transitions of Real and Model Ecosystems.

[40] Snyder W.E. (2019). Give predators a complement: Conserving natural enemy biodiversity to improve biocontrol. Biol. Control.

[41] Morris R.J., Lewis O.T., Godfray H.C.J. (2004). Experimental evidence for apparent competition in a tropical forest food web. Nature.

[42] Müller C., Adriaanse I., Belshaw W., Godfray H. (1999). The structure of an aphid-parasitoid community. J. Anim. Ecol..

[43] Gagic V., Tscharntke T., Dormann C.F., Gruber B., Wilstermann A., Thies C. (2011). Food web structure and biocontrol in a four-trophic level system across a landscape complexity gradient. Proc. R. Soc. B Biol. Sci..

[44] Lohaus K., Vidal S., Thies C. (2013). Farming practices change food web structures in cereal aphid-parasitoid-hyperparasitoid communities. Oecologia.

[45] Rosenheim J.A. (1998). Higher-order predators and the regulation of insect herbivore populations. Annu. Rev. Entomol..

[46] Thies C., Roschewitz I., Tscharntke T. (2005). The landscape context of cereal aphid-parasitoid interactions. Proc. R. Soc. B Biol. Sci..

[47] Gómez-Marco F., Urbaneja A., Jaques J.A., Rugman-Jones P.F., Stouthamer R., Tena A. (2015). Untangling the aphid-parasitoid food web in citrus: Can hyperparasitoids disrupt biological control?. Biol. Control.

[48] Alvarez-Baca J.K., Montealegre X., Le Lann C., Van Baaren J., Lavandero B. (2022). Effect of a cover crop on the aphid incidence is not explained by increased top-down regulation. PeerJ.

[49] Montes C., Perez-Quezada J.F., Peña-Neira A., Tonietto J. (2012). Climatic potential for viticulture in Central Chile. Aust. J. Grape Wine Res..

[50] Sarricolea P., Herrera-Ossandon M., Meseguer-Ruiz Ó. (2017). Climatic regionalisation of continental Chile. J. Maps.

[51] DGAC Reporte Climático 2018. www.meteochile.gob.cl.

[52] Blackman R., Eastop V. (2007). Taxonomic Issues.

[53] Blackman R., Eastop V. (2000). Aphids on the World’s Crops: An Identification and Information Guide.

[54] González R. (1989). Insectos y Acaros de Importancia Agricola y Cuarentenaria en Chile.

[55] Colfer R.G., Rosenheim J.A. (2001). Predation on immature parasitoids and its impact on aphid suppression. Oecologia.

[56] Nieto Nafría J.M., Fuentes-Contreras E., Castro Colmenero M., Aldea Piera M., Ortego J., Mier Durante M.P., Durante M.P.M. (2016). Catálogo de los áfidos (Hemiptera, Aphididae) de Chile, con plantas hospedadoras y distribuciones regional y provincial. Graellsia.

[57] Starý P. (1995). The Aphidiidae of Chile (Hymenoptera, Ichneumonoidea, Aphidiidae). Dtsch. Entomol. Z..

[58] Tomanovic Ž., Petrovic A., Mitrovic M., Kavallieratos N., Stary P., Rakhshani E., Rashanipour M., Popovic A., Shukshuk A., Ivanovic A. (2014). Molecular and morphological variability within the *Aphidius colemani* group with redescription of *Aphidius platensis* Brethes (Hymenoptera: Braconidae: Aphidiinae). Bull. Entomol. Res..

[59] Hullé M., Chaubet B., Turpeau E., Simon J.C. (2020). Encyclop’Aphid: A website on aphids and their natural enemies. Entomol. Gen..

[60] Folmer O., Black M., Hoeh W., Lutz R., Vrijenhoek R. (1994). DNA primers for amplification of mitochondrial cytochrome C oxidase subunit I from diverse metazoan invertebrates. Mol. Mar. Biol. Biotechnol..

[61] Hall T.A. (1999). BioEdit: A user-friendly biological sequence alignment program for Windows 95/98/NT. Nucleic Acids Symp. Ser..

[62] Sint D., Traugott M. (2016). Food Web Designer: A flexible tool to visualize interaction networks. J. Pest Sci..

[63] Dormann C.F., Frund J., Bluthgen N., Gruber B. (2009). Indices, graphs and null models: Analyzing bipartite ecological networks. Open Ecol. J..

[64] Maunsell S.C., Kitching R.L., Burwell C.J., Morris R.J. (2015). Changes in host-parasitoid food web structure with elevation. J. Anim. Ecol..

[65] Beckett S., Devoto M., Felix G., Iriondo J., Op-Sahl T., Pinheiro R., Strauss R., Vazquez D., Clauset A., Rodriguez M. (2020). Visualising Bipartite Networks and Calculating Some (Ecological) Indices.

[66] Oksanen F.J., Simpson G., Blanchet F., Kindt R., Legendre P., Minchin P., O’Hara O., Solymos P., Henry M., Stevens H. (2017). Vegan: Community Ecology Package. R package Version 2.4-3. https://cran.r-project.org/package=vegan.

[67] R Core Team (2019). R: A Language and Environment for Statistical Computing.

[68] Bates D., Mächler M., Bolker B., Walker S. (2015). Fitting Linear Mixed-Effects Models Using lme4. J. Stat. Softw..

[69] Fox J., Weisberg S., Adler D., Bates D., Baud-Bovy G., Ellison S., Firth D., Friendly M., Gorjanc G., Graves S. (2016). Package “Car”. Companion to Applied Regression Depends R (>=3.2.0).

[70] Hothorn T., Bretz F., Westfall P. (2008). Simultaneous inference in general parametric models. Biom. J..

[71] Ortiz-Martínez S.A., Lavandero B. (2018). The effect of landscape context on the biological control of *Sitobion avenae*: Temporal partitioning response of natural enemy guilds. J. Pest Sci..

[72] Poelman E.H., Cusumano A., De Boer J.G. (2022). The ecology of hyperparasitoids. Annu. Rev. Entomol..

[73] Sullivan D.J., Völkl W. (1999). Hyperparasitism: Multitrophic ecology and behavior. Annu. Rev. Entomol..

[74] Buitenhuis R., McNeil J.N., Boivin G., Brodeur J. (2004). The role of honeydew in host searching of aphid hyperparasitoids. J. Chem. Ecol..

[75] Nakashima Y., Higashimura Y., Mizutani K. (2016). Host discrimination and ovicide by aphid hyperparasitoids *Asaphes suspensus* (Hymenoptera: Pteromalidae) and *Dendrocerus carpenteri* (Hymenoptera: Megaspilidae). Appl. Entomol. Zool..

[76] Sullivan D.J. (1987). Insect hyperparasitism. Annu. Rev. Entomol..

[77] Albrecht M., Duelli P., Schmid B., Müller C.B. (2007). Interaction diversity within quantified insect food webs in restored and adjacent intensively managed meadows. J. Anim. Ecol..

[78] Zepeda-Paulo F.A., Ortiz-Martínez S.A., Figueroa C.C., Lavandero B. (2013). Adaptive evolution of a generalist parasitoid: Implications for the effectiveness of biological control agents. Evol. Appl..

[79] Lérault L., Clavel E., Villegas C.M., Cabrera N., Jaloux B., Plantegenest M., Lavandero B. (2022). Providing alternative hosts and nectar to aphid parasitoids in a plum orchard to determine resource complementarity and distance range effect on biological control. Agronomy.

[80] Veres A., Petit S., Conord C., Lavigne C. (2013). Does landscape composition affect pest abundance and their control by natural enemies? A review. Agric. Ecosyst. Environ..

[81] Dong Z., Men X., Liu S., Zhang Z. (2019). Food web structure of parasitoids in greenhouses is affected by surrounding landscape at different spatial scales. Sci. Rep..

[82] Van Veen F.J.F., Müller C.B., Pell J.K., Godfray H.C.J. (2008). Food web structure of three guilds of natural enemies: Predators, parasitoids and pathogens of aphids. J. Anim. Ecol..

[83] Raymond L., Plantegenest M., Gagic V., Navasse Y., Lavandero B. (2016). Aphid parasitoid generalism: Development, assessment, and implications for biocontrol. J. Pest Sci..

[84] Philpott S.M., Lucatero A., Bichier P., Egerer M.H., Jha S., Lin B., Liere H. (2020). Natural enemy–herbivore networks along local management and landscape gradients in urban agroecosystems. Ecol. Appl..

